# Determinants of Dribbling and Passing Skills in Competitive Games of Women’s Basketball

**DOI:** 10.3390/ijerph18031165

**Published:** 2021-01-28

**Authors:** Tomáš Vencúrik, Jiří Nykodým, Dominik Bokůvka, Tomislav Rupčić, Damir Knjaz, Vedran Dukarić, Ivan Struhár

**Affiliations:** 1 Department of Sports, Faculty of Sports Studies, Masaryk University, 62500 Brno, Czech Republic; nykodym@fsps.muni.cz (J.N.); bokuvka@fsps.muni.cz (D.B.); 2 Laboratory for Sports Games, Faculty of Kinesiology, University of Zagreb, 10000 Zagreb, Croatia; tomislav.rupcic@kif.hr (T.R.); damir.knjaz@kif.unizg.hr (D.K.); vedran.dukaric@kif.hr (V.D.); 3 Department of Health Promotion, Faculty of Sports Studies, Masaryk University, 62500 Brno, Czech Republic; struhar@fsps.muni.cz

**Keywords:** performance analysis, offensive skills, effectivity, disruptive factors

## Abstract

This work aimed to identify the influence of selected endogenous (internal load) and exogenous (possession duration, game quarter, and defensive pressure) factors in natural game conditions on the efficiency of dribbling and passing skills. Dribbling and passing skills were assessed during four games of U19 female basketball players and five games of senior (2nd division) female basketball players. In total, 551 dribbling and 914 passing executions were evaluated. Binary logistic regression identified defensive pressure of the opponent as a predictor of dribbling and passing skills effectivity. When the defensive pressure of the opponent was medium, the chances for the ineffective pass were 1.997 times more likely (95% CI; 1.179–3.383), as it is at the minimum defensive pressure. When the defensive pressure of the opponent was high, the chances for ineffective dribbling were 7.45 times more likely (95% CI; 3.672–15.113) and for ineffective pass were 8.419 times more likely (95% CI; 4.6–15.409), as it is at minimum defensive pressure. The game quarter and the internal load were not identified as the predictors influencing the dribbling and passing effectivity. Possession duration was also an insignificant predictor of dribbling and passing skills effectivity. However, the passing skill effectivity decreases when the shot clock is winding down. These findings confirm the importance of transferring game situations into the training process. Coaches should take into account these factors when they want to stimulate determinants of player performance in a balanced and systematic way.

## 1. Introduction

Analysis of the game and knowledge of player performance (PP) in basketball and its structure has both theoretical and practical significance. In theoretical terms, it is an explanation of the essence of sports performance phenomenon. In practical terms, it is forming conclusions about the construction, organization, and management of the training process. Getting into the structure of PP as a multifactorial phenomenon is very difficult due to the many factors that affect it. According to Hughes and Bartlett [1], its quality can be indirectly assessed by performance indicators. These indicators provide metric properties for objectively monitored PP characteristics on specific numerical scales, thus allowing valid interpretation of player performance [2].

PP in a game always takes place under the influence of various disruptive factors. Resistance to disruptive factors is understood as a player’s ability to perform a given game activity (to solve movement tasks) without reducing its effectiveness. Therefore, disruptive factors that result from endogenous factors (intense muscular work, emotional tension, etc.) and exogenous factors (situational unexpectedness, defensive pressure, etc.) reduce the player’s performance in the game. These actions have a disruptive effect on various systems of the organism, enter into mental processes, increase the requirements for energy coverage of physical activities, and can cause biomechanical barriers in movement performance.

Csataljay et al. [3] observed in their study the influence of the defender’s physical pressure on the successfulness of basketball shooting under the game conditions. Their findings confirmed the general premise that the increasing level of physical pressure of the defender had a significant impact on reducing the percentage of shooting successfulness and on the winning the game. Lyons et al. [4] tried to model the intensity of the game load and determine its impact on the accuracy of passing (standardized basketball test). They succeeded in confirming that with increasing load intensity and increasing fatigue, the accuracy of passing decreases. In similar research [5], documented the effect of increasing load intensity on the kinematics of shooting from 7.24 m (3-point arc distance in NBA). They state that due to the high load intensity, there were significant changes in the shooting angles of the individual arm segments, which in turn influenced the shooting successfulness from this distance. Leite et al. [6] found the effect of the physical pressure of the defender and Sampaio et al. [7] in turn, the impact of the load intensity on the movement behavior of basketball players in the offensive phase of the game (an indicator of the team’s tactical side). According to the study by Refoyo et al. [8], in game-based drills, the increasing load intensity and increasing physical pressure of the defender has a significant impact on the correctness of decision-making in the offensive phase of the game. Gorman and Maloney [9] reported a decrease in shooting accuracy of over 20% when players shot with the presence of defender.

Based on the studies above, it can be concluded that these disruptive factors significantly reduce the quality of PP. In this respect, however, there is a considerable deficiency in this area—most of the studies assess the impact of disruptive factors: (i) On male player performance; (ii) under modelled or training conditions. There is also a selection of individual factors, while their effect should be examined in a complex form, that is, by their simultaneous influence on PP. Only a few studies have addressed the influence of disruptive factors on the effectiveness of shooting in males [3,10] but not on the effectiveness of other offensive player skills such as dribbling and passing and even in females. The study aimed to identify the predictors of dribbling and passing effectivity in female basketball players’ natural game conditions. We hypothesized that dribbling and passing effectivity would be affected by selected endogenous (internal load) and exogenous (possession duration, game quarter, and defensive pressure) factors.

## 2. Materials and Methods

### 2.1. Participants

The research group consisted of a female basketball team playing in the 1st division (the highest competition in the Czech Republic) of the U19 age category (*n* = 14) and a female basketball team playing in the 2nd division in the senior category (*n* = 12). Since these were competitive games, the played time of the players’ as well as the line-up and substitutions could not be influenced. The coaches of both teams substituted the players to the best of their belief. Because some players did not meet the inclusion criteria (20 min played and start in both halves), their data were not processed (4 players in the U19 team and 4 players in the team playing in the 2nd division in the senior category). Overall, data from ten elite U19 players (*n* = 10) and eight semi-elite senior team players (*n* = 8) were processed.

Characteristics of U19 basketball players: The average calendar age = 17.6 ± 1 years; the average sports age = 7.4 ± 1.5 years; the average body height = 179.4 ± 6.2 cm; the average body weight = 62.9 ± 5.3 kg. From the point of view of the players’ positions, there were 3 guards, 3 forwards, and 4 centers. Players completed 5–6 training units of 1.5–2 h per week (strength and conditioning + technical–tactical training) and every other weekend 2 competitive games. Characteristics of basketball players of the 2nd division of senior category: The average calendar age = 20 ± 2.8 years; the average sports age = 10 ± 3.2 years; the average body height = 179.8 ± 4.9 cm; and the average body weight = 66.8 ± 5.7 kg. Game positions were represented as follows: 2 guards, 3 forwards, and 3 centers. During the weekly cycle, basketball players underwent 4–5 training units (strength and conditioning + technical–tactical training) and every other weekend 2 competitive games.

Team coaches and basketball players were acquainted with the purpose of the study. All players signed informed consent (in the case of a minor a legal representative) to which they voluntarily agreed to participate in the research and to process the data anonymously. The study was conducted in accordance with the Declaration of Helsinki and following the ethical standards of Masaryk University.

### 2.2. Procedure

The effectiveness of individual offensive player skills (PS) was evaluated in the U19 and 2nd division of women’s age category games given the effect of endogenous and exogenous factors (we called them disruptive factors). The influence of internal load, defensive pressure, possession duration and game quarter on the execution of offensive dribbling and passing skills was monitored. To assess the executions of offensive PS in the offensive phase of the game (when the player is in possession of the ball), we used the standardized Basketball Offensive Game Performance Instrument (BOGPI) [11]. The evaluation procedure is described in the next chapter.

The study has an observational character with monitoring heart rate (HR) because it aimed to identify dribbling and passing effectivity predictors in the natural game conditions. Researchers did not intervene in both teams’ training process at the end of the regular season nor playoff. The players were monitored for two months in fourteen consecutive games. U19 basketball players were monitored in total in five games (two regular season and three playoffs), basketball players playing the 2nd division of women’s age category in nine games (four regular season and five playoffs). U19 players played three games on home-court and two games on the road. Players of the 2nd division of women’s age category played four games on home-court and five games on the road. All games were recorded by a video camera (live stream) operated by the cameraman, and the recordings are stored and freely available on the internet server.

There have been instances where technical failures were unfortunately on the opponents’ courts that made it impossible to work with the recordings (e.g., poor camera placement, poor recording quality, or incomplete recording). In the U19 category, it was one game, but in the 2nd division of women’s age category, it was four games that could not be processed or be included in the results section. In total, four U19 games and five of the 2nd division of women’s age category games were watched.

Two experienced referees officiated each game, the court had a standard size of 28 × 15 m, and the playing time was divided into four 10-min quarters. The breaks between the 1st and 2nd quarters and between the 3rd and 4th quarters lasted two minutes, the half-time between the 2nd and 3rd quarters lasted fifteen minutes.

At the beginning of the study, the monitored basketball players underwent a beep test to determine the individual maximum heart rate (HR_max_) achieved at the end of the test [12]. Basketball players were sufficiently motivated by the coaches to perform their best in the test. Based on HR_max,_ we were able to determine the individual HR zones [13]. In observed games, the players’ HR was monitored. A commercially available Suunto Team Pack telemetry system (Suunto Oy, Vantaa, Finland) was used to monitor HR. The system is standardly used in basketball research (competitive games and practice) [14,15,16,17,18]. The validity of the system was verified in a study by Bouillod et al. [19]. The telemetry device included HR sensors with their own internal memory (Suunto Memory Belts), which were set to measure HR at 2-s intervals, and was synchronized with live playing time (start of the game). The obtained data were further evaluated in the appropriate Suunto Training Manager software program (Suunto Oy, Vantaa, Finland). When monitoring HR during games, we experienced technical problems with HR sensors. For some basketball players, even after downloading the data, HR records were incomplete, which made it impossible to process and analyze them.

### 2.3. Data Collection and Data Processing

Dartfish TeamPro 6.0 software (Dartfish, Fribourg, Switzerland) was used for the notational analysis of monitored games. A standardized Basketball Offensive Game Performance Instrument (BOGPI) [11] was used to assess the effectiveness of the execution of individual player skills. In this standardized instrument, we decided to focus on the indicators that are characteristic of the player in possession of the ball. BOGPI is designed to observe and encode player behavior based on video analysis. The instrument was constructed on the methodology principles of the Game Performance Assessment Instrument (GPAI) and the Team Sport Assessment Procedure (TSAP) [20,21,22,23]. Offensive player skills in this context are dribbling and passing. The effectiveness of the performed offensive PS was transformed into a dichotomous dependent variable. Qualitative character 1 was a code for an effectively executed offensive PS, and qualitative character 0 was a code for an ineffective performed offensive PS (Table 1). A total of 551 dribbling and 914 passing executions were evaluated.

Each execution of offensive PS was assigned codes of selected disruptive factors (independent variables) that could affect their effectiveness (Table 2). The defensive pressure on the player in possession of the ball was classified based on several studies [8,10,24] as: (i) The minimum physical pressure on the player in possession of the ball, (ii) the medium physical pressure on the player in possession of the ball, and (iii) the maximum physical pressure on the player in possession of the ball. Execution of PS has been assigned ball possession duration [25,26,27] to: (i) 0–8 s, (ii) 9–16 s, (iii) 17–24 s. Next factor was game quarter [10,28]: (i) First, (ii) second, (iii) third, and (iv) forth. HR was studied as an endogenous factor. All HR values are related to HR_max_, which respects the individual characteristics of individual basketball players [13]. Based on HR_max_, three HR zones [29,30] were classified: (i) Zone 1: <85% HR_max_ (low to medium intensity), (ii) zone 2: 85–95% HR_max_ (high intensity), (iii) zone 3: >95% HR_max_ (maximal intensity). The HR zones was determined based on the calculation of the mean HR, five seconds before the start of PS execution for the next ten seconds from the beginning of PS execution (total of 15 seconds).

In situations when the basketball player passed accurately (effectively) but the receiving player had difficulty in processing the ball, performing the offensive PS of passing was rated as effective. If a basketball player was fouled by the defender while performing any offensive PS, the execution was rated as effective [10].

Objectivity and reliability. Since we used the method of notational analysis, it was necessary to ensure the objectivity of the evaluation (inter-rater agreement). Three independent expert observers assessed a total of 157 randomly selected PS executions (more than 10%). The following criteria were applied for the selection of professional observers: At least 10 years of basketball coaching experience (youth and seniors, different performance categories); at least 10 years’ experience as a university researcher or lecturer of basketball; the highest coaching education or qualification at a national level; international FIBA Coaching License. The observers were trained and instructed on the procedure of coding the execution of PS.

The reliability (intra-rater agreement) was ensured by repeated assessment of 157 randomly selected PS executions (more than 10%). The intra-individual difference in the evaluation of the PS execution was verified by the researcher at two different time points. The time difference between the first and second assessment was 12 weeks, to exclude the possibility of memorizing the rating of individual game situations [2].

### 2.4. Statistical Analysis

The inter-rater agreement of this nominal dichotomic classification (PS execution) was expressed based on the kappa coefficient (*κ*). The Cohen kappa coefficient was used to determine the agreement in the PS executions between every two observers, while the Fleiss kappa coefficient was used to determine the agreement in the PS executions among all three observers [31]. Krippendorff’s alpha (KALPHA) and its 95% confidence intervals (CI) were used to assess the defensive pressure of inter-rater agreement since in this case, the ordinal-type features were used [32]. The Cohen kappa coefficient was also used to determine intra-individual differences in the evaluation of offensive PS in repeated assessment (reliability). Kappa coefficient values are interpreted as [33]: <0.00 = poor agreement; 0.00–0.20 = slight agreement; 0.21–0.40 = fair agreement; 0.41–0.60 = moderate agreement; 0.61–0.80 = substantial agreement; 0.81–1.00 = almost perfect agreement. In the repeated assessment of the level of defensive pressure at two different time points, KALPHA was also used to determine intra-individual differences. The calculated KALPHA can take values from 0 to 1, where 0 means no agreement between observers and 1 means an absolute agreement between observers.

The effects between PS execution and independent variables (defensive pressure, possession duration, game quarter, and internal load) was captured in pivot tables and expressed by Pearson chi-square (*χ*^2^) test. Effect size (*ES*) was calculated by Cramer contingency coefficient and interpreted as: 0.10 = small effect; 0.30 = medium effect; 0.50 = large effect.

For the prediction of the dependent variable (PS effectivity) based on a set of independent variables (defensive pressure, possession duration, game quarter, internal load) the method of binary logistic regression was used [34,35]. The maximum likelihood method was used to estimate regression coefficients. We used the likelihood ratio to interpret the parameters of the logistic regression model. For this study, the independent variables were determined as categorical and were replaced by dummy variables that were re-coded by the statistical software. A reference category has been appointed for each category. The reference category was low pressure, 0–8 s, first quarter and zone 1 (<85% HR_max_) for defensive pressure, possession duration, game quarter and internal load, respectively. The likelihood ratio was, therefore expressed in relation to the reference category [34]. Using backward stepwise selection, we sought for a logistic regression model that best describes the data. The likelihood ratio test verified the logistic regression model. The Wald’s test verified the statistical significance of the regression coefficients, and 95% confidence intervals were constructed for the likelihood ratio. The Hosmer–Lemeshow goodness of fit test was used to determine the difference between observed and expected frequencies.

All statistical tests were evaluated at the level of statistical significance *α* = 0.05, and the statistical software IBM SPSS Statistics 25 (IBM Corp., New York, NY, USA) and Statistica 12 (StatSoft, Inc., Tulsa, OK, USA) was used for their calculation.

## 3. Results

### 3.1. Objectivity and Reliability

Table 3 shows the values of the selected coefficients. All calculated values of the coefficients indicate an almost perfect agreement in inter-rater and intra-rater observations.

### 3.2. Effects between Variables

**Figure 1:**
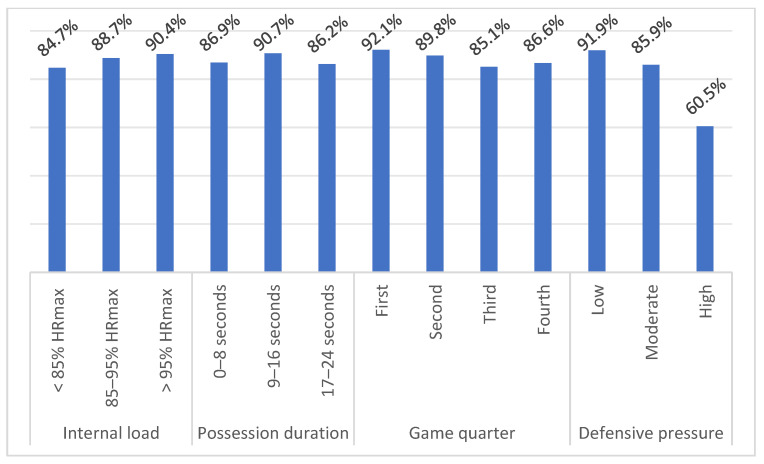
Effectivity of dribbling skill according to independent variables.

**Figure 2:**
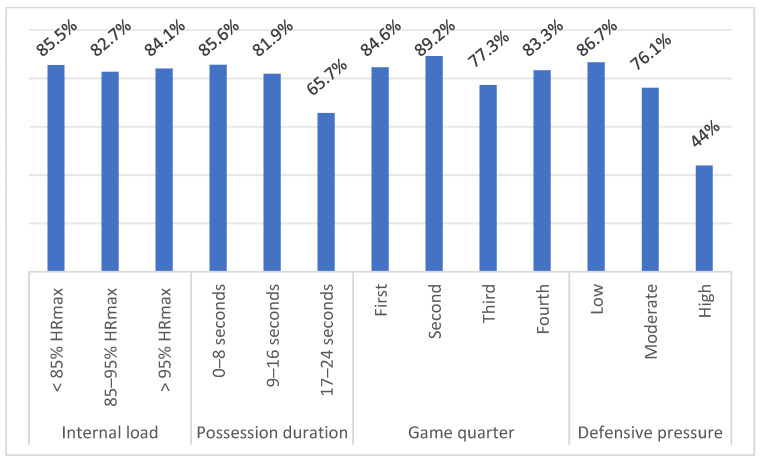
Effectivity of passing skill according to independent variables.

Table 4 and Table 5 shows the sample distribution of each independent variable for the effectiveness of dribbling and passing skills. The tables also include effects between variables based on Pearson’s chi-square test. There is no effect between the internal load and effectivity of dribbling and passing. Possession duration did not affect the effectiveness of dribbling, but on the other hand, it affected the effectiveness of passing. With the time winding down, in the offense, the effectiveness of passing decreased (0–8 s = effectivity 85.6%; 9–16 s = effectivity 81.9%; 17–24 s = effectivity 65.7%). The dependency between the game quarter and dribbling effectiveness was statistically insignificant; however, the effect between the game quarter and the effectiveness of the passing points to statistical significance. The lowest effectivity was recorded in the 3rd quarter (77.3%). The effectivity of dribbling and passing had a decreasing tendency with increasing defensive pressure—91.9%, 85.9%, and 60.5% for dribbling, and 86.7%, 76.1%, and 44% for passing for minimum, average, and maximum defensive pressure, respectively. The effectiveness of dribbling and passing with respect to disruptive factors is shown in Figure 1 and Figure 2.

### 3.3. Variables Determining Ineffectiveness

The prediction of dribbling and passing ineffectiveness was modelled by binary logistic regression, based on independent variables (predictors). Backward stepwise selection, in dribbling, removed insignificant predictors from the model in 4 steps based on the likelihood ratio test (Chi-square = 28.025, df = 2, *p* < 0.001). The adjusted model with one independent variable classified the correct executions of dribbling to 88.4%. For passing, insignificant predictors were removed in 3 steps (Chi-square = 58.777, df = 5, *p* < 0.001). The adjusted model with two independent variables classified the correct execution of the passing to 84%. The estimation of the parameters of the regression models in the last step with explanatory variables, their standard errors and the odds ratio with confidence intervals is given in Table 6. Based on the results, it can be stated that the independent variable that significantly affects dribbling execution is defensive pressure. The independent variables that significantly affect the execution of the passing are the defensive pressure and the quarter. However, when interpreting the estimated parameters, we are limited by taking into account the odds ratio with respect to the reference category that was chosen at the beginning of the analysis. The chance of ineffective dribbling execution at the defender’s maximum physical pressure is 7.45 times more likely (95% CI; 3.672–15.113), compared to the minimum pressure. The chance of ineffective passing execution is, at the defender’s average physical pressure, 1.997 times more likely (95% CI; 1.179–3.383), as it is at the minimum physical pressure. The chance of ineffective passing execution at the defender’s maximum physical pressure is 8.419 times more likely (95% CI; 4.600–15.409) as it is at minimum physical pressure. No statistical differences were found for the quarter concerning the reference category. The Hosmer–Lemeshow test indicates a good fit in the fourth and the third step. The difference between the observed frequencies and the expected frequencies is small, and therefore, not statistically significant.

## 4. Discussion

### 4.1. Internal Load

The Pearson Chi-square test did not confirm the effect between the internal load and the execution of dribbling and passing. Logistic regression identified internal load as an insignificant predictor of dribbling and passing. As many as 86.9% of dribbling executions and 84.9% of passing executions were performed at HR above the level of 85% of HR_max_. Refoyo et al. [8] found a statistically significant effect between PS realization (in game-based drills 1 vs. 1, 2 vs. 2, 2 vs. 1, 3 vs. 2) and the internal load. At low intensity, players were successful at 68.1%, at the average intensity at 62.2%, and at high intensity at 61.6%. However, Refoyo et al. [8] classify the internal load below the aerobic threshold, between the aerobic and anaerobic thresholds, and above the anaerobic threshold. The percentage success of dribbling and passing was lower than in our research: 84.7% (<85% of HR_max_), 88.7% (85–95% of HR_max_), 90.4% (>95% of HR_max_) for dribbling; and 85.5% (<85% of HR_max_), 82.7% (85–95% of HR_max_), 84.1% (>95% of HR_max_) for passing. The difference in successfulness can probably be caused by different categorization of internal load. Vencúrik [36] reports a shooting success rate of 60%, 37.5%, and 45.16% for internal load <85% of HR_max_, 85–95% of HR_max_, and >95% of HR_max_, respectively. In the study mentioned above, the author did not find effect between the successfulness of the shooting and the internal load, which corresponds with our results. On the other hand, in study of Padulo et al. [37], the effectivity of 2 pt shooting with HR above 80% of HR_max_ (30%) is significantly lower compared to the effectivity of shooting with HR at 50% of HR_max_ (38.2%) and at rest (42.3%). Similar results showed a study of Ardigo et al. [38], where effectivity of 3 pt shooting was lower with HR above 80% of HR_max_ (36.8%) when compared with the effectivity with HR at 50% of HR_max_ (41.3%) and at rest (46.8%). However, these studies evaluated only the effectivity of shooting with respect to the internal load in training conditions.

The influence of load intensity on the kinematic parameters of long-distance shooting was explained by Erčulj and Supej [5]. They state that due to the high intensity, there were significant changes in the shooting angles of individual arm segments, which subsequently affected the successfulness of shooting from this distance. These findings document the fact that the difference in the successfulness of the execution of PS may vary depending on external conditions (test vs. game), which also confirmed Sachanidi et al. [39] in their study. The test evaluates comprehensively one PS, while in the game usually occur PS chains, and other factors in the context of the game must be taken into account. The insignificance of the influence of the internal load on the execution of dribbling and passing could probably be the result of good fitness readiness of basketball players, and automation of PS in the discomfort of higher internal load. On the other hand, interpretation of the heart rate related to the internal load during competitive games must be cautious because heart rate could be affected by various factors [17].

### 4.2. Possession Duration

The effectiveness of dribbling was approximately at the same level in each time interval of ball possession: 86.9% (0–8 s), 90.7% (9–16 s), and 86.2% (17–24 s). Passing effectiveness decreased with the shot clock winding down in the offense by 85.6% (0–8 s), 81.9% (9–16 s), and 65.7% (17–24 s). The effect between possession duration and the execution of the passing was statistically significant but based on logistic regression; it is an insignificant predictor. Studies by Gómez et al. [27] and Vaquera et al. [26] point to a positive effect between ball screen effectiveness and ball possession. The cause may be disorganization of the defense and more significant fatigue of the defensive players, especially when two or more players cooperate in the offense [40,41]. On the other hand, if the defenders are well organized even at the end of the ball possession, then the offensive players may come under greater psychological pressure with time winding down, and this may also affect their decision-making processes and the effectiveness of passing.

### 4.3. Game Quarter

Dribbling and passing efficiency were lower in the 3rd and 4th quarter compared to the 1st and 2nd. The dependency between the quarter and the execution of passing was statistically significant, and the logistic regression identified the quarter as a significant predictor. However, no statistically significant differences to the reference category were found. Vaquera et al. [26] did not find a significant impact of the game quarter to ball screen effectiveness. In our case, the lower efficiency of the passing in the 3rd quarter could be caused by the fact that in the half-time break the players have new tactical instructions, which may culminate in greater pressure in defense. Even if the difference in score is too significant, players are highly motivated to still do something about the result of the match.

### 4.4. Defensive Pressure

The effectiveness of dribbling and passing had a declining trend with increasing physical pressure. At the high defensive pressure, the efficiency of execution was 60.5% and 44%, for dribbling and passing, respectively. The effect between physical pressure and dribbling and passing was statistically significant. Logistic regression identified the defensive pressure as a significant predictor of inefficient execution of dribbling and passing. The chance of unsuccessful dribbling was at the defensive pressure 7.45 times more likely compared to the minimum defensive pressure. The chance at the average defensive pressure compared to the minimum defensive pressure was 1.997 times more likely, while at the maximum defensive pressure compared to the minimum defensive pressure up to 8.419 times more likely. Our results confirmed the general belief of the coaches that the chance of successful execution of the PS is significantly reduced by a maximum defensive pressure [42]. The same results are presented by Csataljay et al. [3], Vencurik and Nykodym [43], and by Gorman and Maloney [9] where they also report a statistically significant effect of increasing defensive pressure on reducing the percentage of shooting successfulness. On the other hand, Gómez et al. [10] do not report the influence of the maximum defensive pressure as a significant predictor of shooting successfulness. These findings may have been caused by differences in gender (men), performance (elite players) and the overall quality of the competition (FIBA Basketball World Cup).

### 4.5. Practical Applications

Passing skills are essential to increase points scored in top-level basketball competitions such as Euroleague or NBA [28,44]. Moreover, when players possessed the ball and faced increased defensive pressure, they selected passing rather than any other action [45]. Based on our study, we know that increasing defensive pressure decreases the effectivity of passing and dribbling. Therefore, dribbling and passing skills should be developed in game-based drills, where a presence of defender and the internal load are similar to the competitive games. For dribbling skills, this could happen in small-sided games with fewer players (e.g., 2 vs. 2) where dribbling frequency is higher [46]. For the development of passing skills are recommended the following: No dribble game drills, game drills with dribble restrictions and small-sided games that allow a high number of passes and interceptions. Our practical applications are in conjunctions with other studies (small-sided games research) [47,48,49], suggesting that coaches could use these drills to train technical actions relevant to young players’ decision-making capabilities in game-based situations. Therefore, it is necessary to look for other endogenous and exogenous factors that could affect the PP to clarify better the nature of PP [50,51,52].

### 4.6. Limitations

First, it may appear to be the research sample. The results of the study should not be generalized for all basketball players. They can be considered valid for female players of the same performance level and age category. The teams in which the monitored basketball players played are among the top teams in their age and performance categories in the Czech Republic. U19 team consisted of the elite basketball players of this age category. Eight players were in the given year a part of the national selection team (U18 and U20). Seven players from the senior team had experience from youth national selection teams and the senior 1st division. Moreover, the senior team won in the monitored season 2nd division and advanced for the next season to 1st division. Therefore, the data obtained from monitoring these elite (semi-elite) players have a referential value, and results may be an asset in the field of basketball science. In similar basketball research of elite (semi-elite) players it is an acceptable number of subjects [53,54,55,56,57,58].

Second, the evaluation of internal load based only on HR can be limiting. The ideal solution would be to express the internal load in addition to the HR also with the values of the maximum oxygen consumption (VO_2_max), blood lactate and the Rate of Perceived Exertion (RPE). Due to the natural conditions of the games, the evaluation of the internal load based on HR was the only possible solution. HR is a valid indicator, which quantifies cardiac response, and it is used to assess the internal load during competitive games and practices. No other physiological parameters than HR are available that provide a non-invasive, time-efficient, cost-effective, and continuous insight into a human’s physiological response in almost any environment. On the other hand, HR could be affected by various factors. Those factors may influence HR responses during basketball training and game-play, which includes internal, environmental, technical, and activity-specific aspects. Therefore, additional information about the internal load should be considered when interpreting changes in the HR [16,17,59,60].

## 5. Conclusions

This study’s novelty lies in the identification of disturbing factors that affect PP in the competitive conditions of female basketball players. By identifying these factors and determining their impact, it is then possible to ensure a better transfer of game conditions into the training process (and vice versa), which should lead to more significant progress in PP.

The training process should stimulate the determinants of individual PP in a balanced and systematic way. From this point of view, the results indicate the importance of determinant, which should be given increased attention in the training process of the monitored age and performance categories. We recommended developing dribbling and passing skills of female basketball players in game-based drills, where a presence of defender and the internal load is similar to the competitive games. The physical pressure of the defender should be in these drills on medium or high level.

By expanding the research sample and implementing a larger number of measurements, we could reach more detailed results. We recommend verifying the results on other age and performance categories of female and male basketball players, which could lead to generally valid conclusions.

## Figures and Tables

**Table 1:** Coding of dribbling and passing execution.

Coding	Execution of Offensive Player Skills	
	Dribbling	Passing
Code 1	- The player does not lose control of the ball while dribbling (with speed changes and changes of direction), - The player handles the ball without visual control, watches teammates and action of the court.	- An accurate pass to the teammate who is open and has no problem catching the ball.
Code 0	- The player has difficulty controlling the ball and loses control of the ball (with speed and direction changes), - The player has to control the ball visually, does not watch the teammates, loses track of what is happening on the court, - The ball is lost while dribbling—travelling.	- The pass is inaccurate, the teammate has a problem catching the ball (low pass, the pass is too high, too far forward or too far back from the teammate) and does not gain an advantage over the opponent. - The pass is inaccurate, and the ball is lost (the ball ends up out of bounds, the defender catches the ball) or the offense deflects it out of bounds.

**Table 2:** Variables and their value.

Variable		Value
Dependent variable	Realization	Effective
		Ineffective
Independent variable	Defensive pressure	Low
		Medium
		High
	Possession duration	0–8 s
		9–16 s
		17–24 s
	Game quarter	First
		Second
		Third
		Fourth
	Internal load	Zone 1: <85% HR_max_
		Zone 2: 85–95% HR_max_
		Zone 3: >95% HR_max_

**Table 3:** Inter-rater and intra-rater agreement in different observations.

Agreement	Observer	Dribbling and Passing		Defensive Pressure	
		Cohen’s Kappa	Fleiss’ Kappa	KALPHA	95% CI
Inter-rater agreement	AxB	0.954	0.855	0.846	0.765–0.921
	AxC	0.789			
	BxC	0.830			
Intra-rater agreement	AxA	0.970	-	0.939	0.874–0.982

Note: KALPHA—Krippendorff’s alpha; CI—confidence intervals.

**Table 4:** Frequency distribution (%) of dribbling skill according to independent variables.

Independent Variables		The Realization of Dribbling Skill		χ^2^	df	*p*	ES
		Effective	Ineffective				
Internal load	<85% HR_max_	61 (11.1%)	11 (2.0%)	1.265	2	0.531	0.048
	85–95% HR_max_	360 (65.3%)	46 (8.3%)				
	>95% HR_max_	66 (12.0%)	7 (1.3%)				
Possession duration	0–8 s	258 (46.8%)	39 (7.1%)	1.940	2	0.379	0.059
	9–16 s	204 (37.0%)	21 (3.8%)				
	17–24 s	25 (4.6%)	4 (0.7%)				
Game quarter	First	139 (25.2%)	12 (2.2%)	4.258	3	0.235	0.088
	Second	114 (20.7%)	13 (2.3%)				
	Third	137 (24.9%)	24 (4.4%)				
	Fourth	97 (17.6%)	15 (2.7%)				
Defensive pressure	Low	376 (68.2%)	33 (6.0%)	38.277	2	<0.001	0.264
	Moderate	85 (15.4%)	14 (2.6%)				
	High	26 (4.7%)	17 (3.1%)				

Note: χ^2^—chi-square; df—degrees of freedom; *p*—statistical significance; ES—effect size.

**Table 5:** Frequency distribution (%) of passing skill according to independent variables.

Independent Variables		The Realization of Passing Skill		χ^2^	df	*p*	ES
		Effective	Ineffective				
Internal load	<85% HR_max_	118 (12.9%)	20 (2.2%)	0.696	2	0.706	0.028
	85–95% HR_max_	564 (61.7%)	118 (12.9%)				
	>95% HR_max_	79 (8.6%)	15 (1.7%)				
Possession duration	0–8 s	421 (46.1%)	71 (7.8%)	10.117	2	0.006	0.105
	9–16 s	317 (34.7%)	70 (7.6%)				
	17–24 s	23 (2.5%)	12 (1.3%)				
Game quarter	First	208 (22.8%)	38 (4.2%)	11.058	3	0.011	0.110
	Second	205 (22.4%)	27 (2.9%)				
	Third	198 (21.7%)	58 (6.3%)				
	Fourth	150 (16.4%)	30 (3.3%)				
Defensive pressure	Low	669 (73.2%)	103 (11.3%)	65.087	2	<0.001	0.267
	Moderate	70 (7.6%)	22 (2.4%)				
	High	22 (2.4%)	28 (3.1%)				

Note: χ^2^—chi-square; df—degrees of freedom; *p*—statistical significance; ES—effect size.

**Table 6:** Estimation of regression model parameters for execution of dribbling and passing with explanatory variables in the last step.

Skill	Independent Variables		B	SE.	Wald	df	*p*	OR	95% CI for OR	
									Lower	Upper
Dribbling	Defensive pressure	Overall			31.099	2	0.000			
		Moderate	0.629	0.341	3.411	1	0.065	1.877	0.962	3.660
		High	2.008	0.361	30.963	1	0.000	7.450	3.672	15.113
Passing	Defensive pressure	Overall			50.381	2	0.000			
		Moderate	0.692	0.269	6.613	1	0.010	1.997	1.179	3.383
		High	2.131	0.308	47.728	1	0.000	8.419	4.600	15.409
	Game quarter	Overall			10.021	3	0.018			
		Second	−0.368	0.280	1.720	1	0.190	0.692	0.400	1.199
		Third	0.444	0.240	3.402	1	0.065	1.558	0.973	2.497
		Forth	0.157	0.276	0.323	1	0.570	1.170	0.681	2.008

Note: B—standardized beta weights; SE—standard error of the estimate; Wald—values of Walds’s test; df—degrees of freedom; *p*—statistical significance of regression coefficients; OR—odds ratio; CI—confidence intervals.

## Data Availability

The data presented in this study are available on request from the corresponding author. The data are not publicly available due to its huge size and participants’ privacy protection.
